# Treatment of Periprosthetic Joint Infection After Tumor Megaprosthetic Reconstruction: A Narrative Review

**DOI:** 10.3390/cancers18020230

**Published:** 2026-01-12

**Authors:** Wei Wang, Haoran Qiao, Zhiqing Zhao, Taiqiang Yan

**Affiliations:** 1 Department of Orthopedics, Peking University First Hospital, Beijing 100034, China; 2011110359@pku.edu.cn (W.W.); zhaozhiqing@pku.org.cn (Z.Z.); 2 Department of Emergency Surgery, Peking University First Hospital, Beijing 100034, China; haoran_qiao@163.com

**Keywords:** megaprosthesis, tumor, periprosthetic joint infection, two-stage revision, limb salvage

## Abstract

**Purpose:** Periprosthetic joint infection (PJI) is a devastating complication following limb salvage surgery with tumor megaprosthetic reconstruction, leading to high morbidity and complex management. Despite advancements in prosthesis design and materials, infection rates are notably higher than in conventional arthroplasty. This narrative review synthesizes current evidence on the etiology, diagnosis, and management of PJIs in this unique setting. **Methods:** We conducted narrative review of literature from PubMed and Embase using keywords related to PJIs and tumor megaprostheses, aiming to summarize risk factors, diagnostic criteria, pathogen profiles, and treatment outcomes. **Results:** Key findings indicate that the risk of PJI is multifactorial, involving patient-related, disease-related, and treatment-related factors. Diagnosis relies on a combination of clinical presentation, serological markers, imaging, and microbiological studies, though established criteria for conventional PJI may require adaptation for tumor cases. Treatment strategies include irrigation and debridement (I&D), debridement, antibiotics, implant retention with modular component exchange (DAIR), one-stage or two-stage revision, and amputation. Success rates vary, and optimal management requires a multidisciplinary, individualized approach. However, two-stage revision is considered the gold standard for chronic PJIs. **Conclusions:** PJIs after tumor megaprosthetic reconstruction presents distinct challenges. Management requires a multidisciplinary, individualized approach. Future research should focus on validated diagnostic criteria for this population, novel anti-biofilm strategies, and standardized treatment protocols.

## Simple Summary

Periprosthetic joint infection (PJI) is a devastating complication, leading to high morbidity and complex management. Reported PJI rates after primary tumor megaprosthetic reconstruction ranged from 5% to 25%, significantly higher than the 0.5% to 2% observed in conventional hip and knee arthroplasty. The management of PJIs after tumor megaprosthetic reconstruction is complex. This narrative review aims to provide a comprehensive and critical summary of the current understanding of PJIs following tumor megaprosthetic reconstruction. It will specifically focus on analyzing the evidence behind risk factors, diagnostic approaches, and treatment outcomes, and will identify persistent knowledge gaps to guide future research and clinical practice.

## 1. Introduction

Limb salvage surgery with tumor megaprosthetic reconstruction is the standard care for restoring bone continuity and joint function after resection of bone tumors around major joints. Its advantages in managing large segmental defects are well-established [1,2]. However, postoperative complications of tumor megaprosthetic reconstruction are notably higher than that in conventional arthroplasty. Henderson et al. recommend categorizing prosthetic failures into five types [3]: Type I: soft tissue failure; Type II: aseptic loosening; Type III: mechanical failure; Type IV: periprosthetic joint infection (PJI); and Type V: local tumor recurrence. Among these failures, PJI being a leading non-oncological cause of failure and amputation (20%) [4].

Reported PJI rates after primary tumor reconstruction ranged from 5% to 25%, significantly higher than the 0.5% to 2% observed in conventional hip and knee arthroplasty [1]. A long-term follow-up study on tumor megaprostheses in 230 patients with malignant bone tumors, with an average follow-up of 29.4 years (range: 25 to 43 years), found that the risk of PJIs did not decrease with time but maintained an annual growth rate of around 1% [5]. The management of PJIs is particularly complex in tumor patients, who often have compromised host defenses due to chemotherapy, extensive surgical dissection, and large implant volumes. Management of PJIs typically requires multiple surgeries, prolonged antibiotic courses, and significantly impacts patients’ quality of life and oncological treatment timelines.

While several published reviews [6,7,8] have addressed PJIs in conventional arthroplasty, the distinct challenges posed by tumor megaprostheses—such as a larger residual cavity, compromised soft tissue coverage, and immunocompromised hosts—warrant a focused discussion. This narrative review aims to provide a comprehensive and critical summary of the current understanding of PJIs following tumor megaprosthetic reconstruction. It will specifically focus on analyzing the evidence behind risk factors, diagnostic approaches, and treatment outcomes, and will identify persistent knowledge gaps to guide future research and clinical practice.

## 2. Methods

This article is a narrative review of the literature concerning periprosthetic joint infection following tumor megaprosthetic replacement. To identify relevant publications, we searched the PubMed and Embase databases using a combination of keywords and Medical Subject Heading terms including “periprosthetic joint infection,” “PJI,” “tumor prosthesis,” “megaprosthesis,” “oncological arthroplasty,” “infection,” “debridement,” “one-stage revision,” “two-stage revision,” and “amputation.” The search was limited to articles published in English, with a focus on literature from the past two decades (2000–2025), though seminal older works were also included. The reference lists of retrieved articles were manually screened for additional relevant studies.

Inclusion criteria were as follows: (1) studies focusing on PJIs following tumor megaprosthetic reconstruction for bone tumors; (2) clinical studies, reviews, or meta-analyses; and (3) articles published in English.

Exclusion criteria were as follows: (1) case reports with fewer than 5 patients; (2) studies focusing solely on conventional arthroplasty PJIs without oncologic context; and (3) non-clinical or purely technical studies.

Given the heterogeneity of the available studies and the narrative nature of this review, a formal systematic review methodology or quality assessment (e.g., PRISMA, GRADE) was not employed. Instead, we adopted a narrative synthesis approach to summarize the current body of evidence, critically appraise key findings, and highlight consensus and controversies.

## 3. Etiology and Risk Factors

According to published studies, the reported incidence of PJIs after primary hip and knee arthroplasty is 0.5% to 2% [9,10,11,12]. The infection rate after primary tumor megaprosthetic reconstruction is even higher, with a meta-analysis showing an average infection rate of 10% after primary tumor joint arthroplasty [13], and is as high as 43% after revision surgery [14,15]. This elevated risk stems from a confluence of factors [16,17,18,19], which can be categorized as follows:

### 3.1. Patient-Related Factors

Patients undergoing tumor resection often present with compromised physiological reserves. Advanced age, malnutrition (anemia, hypoalbuminemia), and comorbidities like diabetes, rheumatoid arthritis, and chronic kidney disease are known risk factors [20,21]. Critically, pre and postoperative chemotherapy induces neutropenia and immunosuppression, significantly heightening infection susceptibility.

### 3.2. Disease-And Surgery-Related Factors

The anatomical location of the tumor profoundly influences PJI risk. For instance, proximal tibia reconstructions carry the highest infection rate (up to 43.3%) due to the poor soft tissue coverage and tenuous blood supply [5]. Pelvic surgeries are prone to contamination from the perineum. The extent of soft tissue and muscle resection required for oncological margins creates a large residual cavity, and devitalized tissue that is ideal for bacterial colonization. Prolonged operative times (>2.5 h), high-volume blood transfusions, and the implantation of large foreign bodies are significant risks [16,17,18]. The use of extendable prostheses in pediatric patients, while addressing limb-length inequality, necessitates multiple surgeries for lengthening, each compounding the infection risk. Postoperative radiotherapy further compromises local tissue viability and healing capacity. Any subsequent surgical procedure on the reconstructed limb, even for wound issues or mechanical failure, increases the cumulative risk of infection.

### 3.3. Classification of PJI

Based on the timing of onset relative to the index surgery, PJI is commonly classified as follows: early (within 2 months), delayed (3 to 24 months), or late (>24 months) [22,23]. This temporal classification helps guide diagnostic suspicion and therapeutic strategy.

## 4. Diagnosis of PJI

There are no validated diagnostic criteria specific to tumor megaprosthesis PJI. While the Musculoskeletal Infection Society (MSIS) and the 2018 evidence-based criteria by Parvizi et al. provide robust frameworks for diagnosing PJI in conventional hip and knee arthroplasty [24], their direct application to tumor cases requires caution. The larger prosthetic surface area, systemic effects of chemotherapy, and altered local anatomy can confound standard diagnostic parameters.

### 4.1. Limitations of Conventional Criteria in Tumor Settings

The systemic inflammatory response from chemotherapy can elevate C-reactive protein (CRP) and the erythrocyte sedimentation rate (ESR) independently of infection. The larger prosthetic surface area may alter synovial fluid dynamics and cell counts. Therefore, while a combination of clinical signs (pain, wound drainage), serology, imaging, and microbiology remains the cornerstone, the thresholds and interpretation may differ. Radiographs may reveal progressive radiolucent lines or a periosteal reaction suggestive of a chronic infection [25]. Advanced imaging like nuclear medicine studies can be helpful but are often non-specific.

### 4.2. Practical Diagnostic Approach

A practical approach to diagnosing a PJI after tumor megaprosthetic reconstruction includes the presence of one or more of the following:

(1) A sinus tract communicating with the prosthesis or direct prosthesis exposure;
(2) Purulent fluid aspirated from the joint or periprosthetic cavity;
(3) A positive microbial culture from at least two separate tissue or fluid samples;
(4) Intraoperative histopathology showing >10 neutrophils per high-power field in multiple fields, indicative of acute inflammation.

### 4.3. Evolving Biomarkers and Techniques

When definitive signs are absent, adjuncts are crucial (Table 1).

Recent research focuses on identifying more reliable biomarkers in synovial fluid. Leukocyte esterase (LE) strips, which detect neutrophil-derived enzymes, offer a rapid, point-of-care test. A positive result suggests inflammation but lacks pathogen specificity. Alpha-defensin, an antimicrobial peptide released by neutrophils in response to pathogens, has shown promising diagnostic accuracy with reported high sensitivity and specificity in standard PJIs [26,27]. Next-generation sequencing (NGS) of synovial fluid can identify pathogen DNA without the need for culture, potentially increasing microbiological yield, especially in culture-negative cases [28,29]. Challenges remain, including cost, potential contamination, and standardization of interpretation.

## 5. Microbiology and Antimicrobial Strategies

The microbiological spectrum of PJIs after tumor megaprosthetic reconstruction is diverse. While coagulase-negative staphylococci and *Staphylococcus aureus* remain predominant (60% to 80%), there is a growing recognition of infections caused by Gram-negative bacilli (e.g., *Escherichia coli*, *Pseudomonas aeruginosa*) and fungi (e.g., Candida species) [26,30]. Infections with these atypical or resistant organisms often present a greater diagnostic and therapeutic challenge.

Obtaining a reliable microbiological diagnosis is paramount. Deep tissue samples obtained during surgery or aspiration are preferred over superficial swabs. Due to the potential for fastidious or slow-growing organisms, cultures should be incubated for at least 2 to 3 weeks. The utility of NGS in culture-negative scenarios is an area of active investigation [28,29].

Antibiotic therapy must be guided by culture and susceptibility results. Pending these results, empiric broad-spectrum therapy covering methicillin-resistant *S. aureus* (MRSA), Gram-negative organisms, and anaerobes is often initiated. For confirmed pathogens, prolonged intravenous followed by oral antibiotic therapy is standard. Common regimens include the following [22]:

(1) MSSA (Methicillin-sensitive *S. aureus*): Beta-lactams (e.g., cefazolin, oxacillin).
(2) MRSA: Vancomycin, daptomycin, or linezolid.
(3) Gram-negative bacteria: Third- or fourth-generation cephalosporins, fluoroquinolones, or carbapenems, based on susceptibility.
(4) Enterococci: Ampicillin or vancomycin, with consideration for synergistic aminoglycosides.
(5) Fungi: Azoles (e.g., fluconazole) or amphotericin B.

The optimal duration of antibiotic therapy, both intravenous and oral, is not standardized for tumor PJIs and is often extrapolated from conventional arthroplasty guidelines, typically ranging from 4 to 6 weeks intravenously, sometimes followed by long-term oral suppression, especially in cases of retained hardware [22].

Some scholars recommend using serological testing as an indicator of treatment response. Previous studies have shown that the sensitivity of synovial fluid neutrophil count is highest in predicting the success of two-stage revision surgery, followed by the erythrocyte sedimentation rate in blood [31,32]. Racano et al. [13] reviewed 4838 patients, and the overall incidence of PJIs was 10%. The use of prophylactic antibiotics was either 30 min before surgery or intraoperatively, with a PJI incidence of 13% when antibiotics were used postoperatively for only 24 h, and 8% for more than 24 h. There is no standard duration for postoperative antibiotic use, ranging from removal of drainage tubes at 0–3–5–7 days. A multicenter prospective study is currently ongoing to determine whether the duration should be 24 h or 5 days [13,33,34].

## 6. Treatment Strategies

The central goal is to eradicate biofilms. The choice of strategy depends on infection chronicity, implant stability, soft tissue status, and pathogen (Table 2). The treatment for PJIs has always been one of the challenges faced by orthopedic surgeons, and mainly includes irrigation and debridement (I&D), debridement, antibiotics, irrigation and implant retention (DAIR), one-stage revision or two-stage revision, arthrodesis, and amputation [35,36]. Biofilm maturity typically occurs around 4 weeks, forming a critical therapeutic window [37]. Therefore, early diagnosis of a PJI is crucial, as a delayed diagnosis leads to the formation of a mature biofilm, resulting in failure of the revision surgery.

### 6.1. Irrigation and Debridement (I&D), and Debridement, Antibiotics, and Implant Retention (DAIR)

I&D and DAIR are limb-salvaging options reserved for patients with acute infections (symptom duration < 4 weeks) who may not tolerate the morbidity associated with revision surgery [23,48]. Success rates are generally low for tumor megaprostheses, and the selection criteria are strict [25,38,39]. Contraindications include the presence of a sinus tract, difficult-to-treat pathogens (e.g., MRSA, fungi), and poor soft tissue conditions [49]. When take I&D into considered, surgeons should consider the option of modular component exchange (DAIR or called DAIR plus). One study in megaprostheses PJIs has compared the treatment success rates in patients treated with I&D and those treated with DAIR [43]. The success rate of standard I&D (50%) was lower than that for DAIR (68%) [43]. Another study conducted by Holzer et al. revealed a 78% success rate for megaprostheses PJI with DAIR [44].

The procedure must be meticulous. It involves radical debridement of all non-viable tissue and the pseudocapsule, with or without exchange of the modular components (e.g., polyethylene liners), and copious pulsed lavage (6 to 9 L) [50]. The use of antiseptic solutions like diluted povidone–iodine as an irrigant has been suggested to improve bacterial eradication [51]. Postoperative intravenous antibiotics are typically administered for 2 to 6 weeks, often followed by long-term oral suppression [23].

### 6.2. One-Stage Revision

One-stage revision involves removal of the infected prosthesis, thorough debridement, and reimplantation of a new prosthesis in a single procedure. Its success in tumor PJIs is also limited, with reported success rates below 50% [18,26]. It may be considered in patients with good soft tissue coverage, known susceptible pathogens, and who are poor candidates for two major surgeries. The use of an antibiotic-loaded polymethyl methacrylate (PMMA) spacer for fixation is common to deliver high local antibiotic concentrations.

### 6.3. Two-Stage Revision

Two-stage revision is the gold standard for managing chronic PJIs (>4 weeks) in North America and is associated with the highest reported success rates (ranged 80% to 90% in non-oncological series, though lower in tumor cases, e.g., 62% to 75%) [18,30,42,46,47]. Recently, the Birmingham Orthopaedic Oncology Meeting gathered 309 experts from 53 countries to debate 20 consensus statements on PJIs; two-stage revision is the gold standard for the management of PJIs and reached a strong consensus [52]. Two-stage revision has a wider range of indications, suitable not only for patients with indications for one-stage revision, but also for those with drug-resistant bacteria, persistent sinuses, and poor soft tissue coverage, as well as patients with diabetes, autoimmune diseases, malignancies, and long-term antibiotic use who may develop fungal or atypical bacterial infections.

The first stage involves removal of all components, radical debridement, and placement of an antibiotic-loaded polymethyl methacrylate (PMMA) spacer. The spacer provides local antibiotic elution and maintains soft tissue tension. A period of organism-specific intravenous antibiotics (typically 4 to 6 weeks) follows. Reimplantation is performed in the second stage after clinical and serological evidence of infection eradication is confirmed. The optimal interval between stages is debated. Trampuz et al. [53] defined a short interval of 2 to 4 weeks and long interval of 6 to 8 weeks until reimplantation. Other authors proposed an interval of 4 to 6 weeks [54] or 9 weeks [55]. However, the reported interval until reimplantation ranged from a few days to several hundred days or even several years, but an interval of around 80 to 100 days is mostly reported while 6 to 8 weeks is common in some regions [55,56,57,58,59,60]. Notably, longer spacer intervals may be associated with reduced infection control after completing a two-stage exchange revision [61].

Challenges specific to tumor two-stage revision include managing large bone defects, achieving stable spacer fixation, and addressing poor soft tissue coverage, which may necessitate flap procedures. Despite its effectiveness, failure rates remain significant, particularly with resistant organisms [62]. Salgado et al. [62] reported a high failure rate of 50% in two-stage revisions in 12 cases of MRSA infections. Ippolito et al. [63] reported a 90% limb salvage rate with two-stage revisions using intramedullary-stabilized spacers, underscoring the potential for success with meticulous technique. To achieve good soft tissue coverage for two-stage revision prostheses, a smaller prosthesis size than the original device may be chosen, the prosthesis stem may be lengthened and thickened, and in some cases, 1 to 2 cm of thin cortical bone may need to be removed due to widened medullary cavities at the prosthesis–bone interface, sometimes requiring the addition of a reinforcement ring or muscle flap coverage.

### 6.4. Amputation

Amputation is a last-resort treatment for uncontrollable infection, massive bone loss, life-threatening sepsis, or when repeated salvage attempts have failed [5,35]. It provides definitive infection control but carries profound functional and psychological consequences. Grimer et al. reported that nearly 30% of patients with infected tumor megaprostheses ultimately required amputation [5]. Jeys et al. reported 98% of them achieved infection-free status when treated with amputation upon PJI diagnosis [18]. Another report showed a 100% success rate after amputation [46]. The decision should be made by a multidisciplinary team in consultation with the patient, weighing the prospects of functional recovery against the burdens of prolonged, potentially unsuccessful salvage attempts.

## 7. Discussion

This review consolidates the unique challenges in diagnosing and managing PJIs following tumor megaprosthetic reconstruction. The key conclusions are as follows:

Diagnosis is Challenging: Standard PJI criteria lack validation in this population. A high index of suspicion and a combination of clinical, serological (interpreted cautiously), and microbiological findings are essential. Emerging tools like alpha-defensin and NGS show promise but require further study in this specific context.

Treatment Must Be Individualized: The choice between I&D, DAIR, one- or two-stage revision, and amputation depends on a multifactorial assessment. For patients with acute PJI with drug-sensitive pathogens, DAIR can be considered and the modular part should be exchanged, as DAIR is associated with a lower failure rate than I&D (Table 2). Two-stage revision remains the most reliable salvage strategy for chronic infections, despite its technical demands and significant failure risk, particularly with resistant organisms like MRSA. In 2020, a systematic review by Nucci et al. analyzing 647 patients with infected tumor megaprostheses reported stark differences in failure rates: DAIR (55.1%), one-stage revision (45.5%), two-stage revision (27.3%), and amputation (2%) [64].

Outcomes Are Inferior to Conventional Arthroplasty PJIs: Due to patient comorbidities, extensive surgery, and implant size, success rates for all salvage procedures are generally lower than those reported for standard joint replacements.

### 7.1. Study Novelty and Clinical Implications

While previous reviews have discussed megaprosthesis complications broadly, this work provides a focused, contemporary synthesis specifically on PJIs, integrating recent data on diagnostic biomarkers and elaborating on the nuanced decision-making between different revision strategies. The proposed diagnostic approach and treatment pathway offer a practical clinical framework.

### 7.2. Limitations

This narrative review has inherent limitations. The included studies are heterogeneous in design, patient population, and outcome definitions, precluding meta-analysis. The evidence is primarily retrospective and observational, with a lack of high-level comparative studies or randomized trials. Therefore, the recommendations and success rates provided should be interpreted as a synthesis of the best available evidence, not as definitive guidelines.

### 7.3. Future Directions and Conclusions

Future efforts should focus on the following:

Developing Validated Diagnostic Criteria: Prospective studies to define and validate PJI diagnostic criteria specific to tumor megaprostheses.

Optimizing Treatment Protocols: Randomized trials comparing antibiotic durations, spacer types (static vs. articulated), and intervals in two-stage revision procedures are urgently needed.

Advancing Prevention and Adjunct Therapies: Research into antimicrobial coatings (silver, iodine) [65,66], local antibiotic delivery systems, and novel anti-biofilm agents (e.g., bacteriophages, dispersin B) holds potential to reduce infection burden. However, evidence for their superiority in revision scenarios is currently lacking [67].

Standardizing Outcome Reporting: Adoption of core outcome sets would facilitate future meta-analyses and improve evidence quality.

## 8. Conclusions

Periprosthetic joint infections remain a formidable complication of tumor megaprosthetic reconstruction, associated with high morbidity and complex decision-making. Successful management hinges on early and accurate diagnosis, which often requires a combination of clinical, serological, and microbiological criteria, with an awareness of the limitations of standard PJI definitions in this unique population. Treatment must be individualized based on infection chronicity, pathogen profile, and local soft tissue conditions. While DAIR may be considered for acute presentations, its success is limited. Two-stage revision remains the benchmark for chronic infections, offering the best chance for infection eradication and limb salvage, though functional outcomes can be compromised. Amputation, while a last resort, is a definitive solution for uncontrollable scenarios. The current literature is marked by heterogeneity and a lack of high-quality comparative studies. There is a pressing need for standardized protocols, multidisciplinary collaboration, and future research focused on improved preventive strategies, diagnostic tools, and reconstructive techniques to improve outcomes for these challenging patients.

## Figures and Tables

**Table 1:** Diagnostic biomarkers for periprosthetic joint infection.

Biomarker	Sample	Principle	Advantages	Limitations in Tumor Setting
CRP/ESR	Blood	Acute-phase reactants	Widely available, inexpensive	Non-specific; elevated by chemotherapy, surgery, tumor
Synovial fluid WBC count and %PMN	Synovial fluid	Direct measure of inflammation	High sensitivity/specificity in conventional PJIs	Cut-offs not validated for megaprostheses; sampling challenges
Leukocyte esterase	Synovial fluid	Detects neutrophil enzymes	Rapid, cheap, point-of-care	Only indicates inflammation, not infection etiology
Alpha-defensin	Synovial fluid	Antimicrobial peptide from neutrophils	High accuracy reported	Expensive; limited validation in immunocompromised hosts
Microbial culture	Tissue/Fluid	Gold standard for pathogen detection	Guides antibiotic therapy	Low sensitivity (biofilm); slow; prior antibiotics affect yield
Next-generation sequencing	Synovial fluid	Detects microbial DNA	High sensitivity, identifies unculturable pathogens	High cost; risk of contamination; unclear clinical utility thresholds

CRP: C-reactive protein; ESR: Erythrocyte sedimentation rate; WBC: White blood cell; PMN: Polymorphonuclear neutrophil.

**Table 2:** Treatment modalities for PJI in tumor megaprostheses: indications and outcomes.

Treatment	Key Indications	Procedure	Reported Success Rate in Tumor Patients	Major Considerations
I&D	Acute infection, well-fixed implant, good soft tissue, with drug-sensitive pathogens	Radical debridement, without exchange of the modular components, antibiotics	16–75% [25,38,39,40,41]	Strict patient selection is critical; high failure rate
DAIR	Acute infection, well-fixed implant, good soft tissue, with drug-sensitive pathogens	Radical debridement, exchange of the modular components, antibiotics	51–78% [4,5,6,7,8,9,10,11,12,13,14,15,16,17,18,19,20,21,22,23,24,25,26,27,28,29,30,31,32,33,34,35,36,37,38,39,40,41,42,43,44,45]	Strict patient selection is critical; high failure rate
One-Stage revision	Chronic infection in patients unfit for two-stage, known susceptible pathogen, good soft tissue	Removal, debridement, and reimplantation in one surgery with antibiotic-loaded cement	45–47% [18,26]	Limited evidence; higher risk of recurrence compared to two-stage.
Two-Stage revision	Chronic infection, gold standard	Stage 1: Removal, debridement, antibiotic spacer. Stage 2: Reimplantation after antibiotics.	62–75% [18,25,26,30,41,42,46,47]	Highest success rate for chronic PJIs; challenges with bone defects/spacer stability; interval between stages debated (often 6–12 weeks).
Amputation	Uncontrollable infection, life-threatening sepsis, massive bone/soft tissue loss, failed salvage	Definitive resection	98–100% [18,41,42,46]	Functional and psychological morbidity; last resort.

I&D: Irrigation and debridement; DAIR: debridement, antibiotics, and implant retention, with modular component exchange.

## Data Availability

No new data were created or analysed in this study. Data sharing is not applicable to this article.

## References

[1] Haijie L., Dasen L., Tao J., Yi Y., Xiaodong T., Wei G. (2018). Implant Survival and Complication Profiles of Endoprostheses for Treating Tumor Around the Knee in Adults: A Systematic Review of the Literature Over the Past 30 Years. J. Arthroplast..

[2] Zhao Z., Yang Y., Yan T., Tang X., Yang R., Guo W. (2021). Outcomes of Fixed-Hinged Knee Prosthesis for Distal Femoral Osteosarcoma in Skeletally Immature Patients: First Results. J. Knee Surg..

[3] Henderson E.R., Groundland J.S., Pala E., Dennis J.A., Wooten R., Cheong D., Windhager R., Kotz R.I., Mercuri M., Funovics P.T. (2011). Failure mode classification for tumor endoprostheses: Retrospective review of five institutions and a literature review. J. Bone Jt. Surg. Am. Vol..

[4] Myers G.J., Abudu A.T., Carter S.R., Tillman R.M., Grimer R.J. (2007). Endoprosthetic replacement of the distal femur for bone tumours: Long-term results. J. Bone Jt. Surg. Br. Vol..

[5] Grimer R.J., Aydin B.K., Wafa H., Carter S.R., Jeys L., Abudu A., Parry M. (2016). Very long-term outcomes after endoprosthetic replacement for malignant tumours of bone. Bone Jt. J..

[6] Kapadia B.H., Berg R.A., Daley J.A., Fritz J., Bhave A., Mont M.A. (2016). Periprosthetic joint infection. Lancet.

[7] Tabaja H., Abu Saleh O.M., Osmon D.R. (2024). Periprosthetic Joint Infection: What’s New?. Infect. Dis. Clin. N. Am..

[8] Nazzal E.M., Herman Z.J., Como M., Kaarre J., Reddy R.P., Wagner E.R., Klatt B.A., Lin A. (2024). Shoulder Periprosthetic Joint Infection: Principles of Prevention, Diagnosis, and Treatment. J. Bone Jt. Surg. Am. Vol..

[9] Jeys L., Grimer R. (2009). The long-term risks of infection and amputation with limb salvage surgery using endoprostheses. Recent. Results Cancer Res. Fortschritte Krebsforsch. Prog. Rech. Sur Cancer.

[10] Koh C.K., Zeng I., Ravi S., Zhu M., Vince K.G., Young S.W. (2017). Periprosthetic Joint Infection Is the Main Cause of Failure for Modern Knee Arthroplasty: An Analysis of 11,134 Knees. Clin. Orthop. Relat. Res..

[11] Li X., Moretti V.M., Ashana A.O., Lackman R.D. (2011). Perioperative infection rate in patients with osteosarcomas treated with resection and prosthetic reconstruction. Clin. Orthop. Relat. Res..

[12] Whitehouse M.R., Parry M.C., Konan S., Duncan C.P. (2016). Deep infection after hip arthroplasty: Staying current with change. Bone Jt. J..

[13] Racano A., Pazionis T., Farrokhyar F., Deheshi B., Ghert M. (2013). High infection rate outcomes in long-bone tumor surgery with endoprosthetic reconstruction in adults: A systematic review. Clin. Orthop. Relat. Res..

[14] Capanna R., Morris H.G., Campanacci D., Del Ben M., Campanacci M. (1994). Modular uncemented prosthetic reconstruction after resection of tumours of the distal femur. J. Bone Jt. Surg. Br. Vol..

[15] Pala E., Trovarelli G., Calabrò T., Angelini A., Abati C.N., Ruggieri P. (2015). Survival of modern knee tumor megaprostheses: Failures, functional results, and a comparative statistical analysis. Clin. Orthop. Relat. Res..

[16] Berbari E.F., Hanssen A.D., Duffy M.C., Steckelberg J.M., Ilstrup D.M., Harmsen W.S., Osmon D.R. (1998). Risk factors for prosthetic joint infection: Case-control study. Clin. Infect. Dis. Off. Publ. Infect. Dis. Soc. Am..

[17] Guo W., Ji T., Yang R., Tang X., Yang Y. (2008). Endoprosthetic replacement for primary tumours around the knee: Experience from Peking University. J. Bone Jt. Surg. Br. Vol..

[18] Jeys L.M., Grimer R.J., Carter S.R., Tillman R.M. (2005). Periprosthetic infection in patients treated for an orthopaedic oncological condition. J. Bone Jt. Surg. Am. Vol..

[19] Morii T., Morioka H., Ueda T., Araki N., Hashimoto N., Kawai A., Mochizuki K., Ichimura S. (2013). Deep infection in tumor endoprosthesis around the knee: A multi-institutional study by the Japanese musculoskeletal oncology group. BMC Musculoskelet. Disord..

[20] Kim E.J., Ha K.H., Kim D.J., Choi Y.H. (2019). Diabetes and the Risk of Infection: A National Cohort Study. Diabetes Metab. J..

[21] McDonald H.I., Thomas S.L., Millett E.R., Nitsch D. (2015). CKD and the risk of acute, community-acquired infections among older people with diabetes mellitus: A retrospective cohort study using electronic health records. Am. J. Kidney Dis. Off. J. Natl. Kidney Found..

[22] Zimmerli W., Trampuz A., Ochsner P.E. (2004). Prosthetic-joint infections. N. Engl. J. Med..

[23] Osmon D.R., Berbari E.F., Berendt A.R., Lew D., Zimmerli W., Steckelberg J.M., Rao N., Hanssen A., Wilson W.R. (2013). Diagnosis and management of prosthetic joint infection: Clinical practice guidelines by the Infectious Diseases Society of America. Clin. Infect. Dis. Off. Publ. Infect. Dis. Soc. Am..

[24] Parvizi J., Tan T.L., Goswami K., Higuera C., Della Valle C., Chen A.F., Shohat N. (2018). The 2018 Definition of Periprosthetic Hip and Knee Infection: An Evidence-Based and Validated Criteria. J. Arthroplast..

[25] Peel T., May D., Buising K., Thursky K., Slavin M., Choong P. (2014). Infective complications following tumour endoprosthesis surgery for bone and soft tissue tumours. Eur. J. Surg. Oncol. J. Eur. Soc. Surg. Oncol. Br. Assoc. Surg. Oncol..

[26] Ercolano L.B., Christensen T., McGough R., Weiss K. (2013). Treatment solutions are unclear for perimegaprosthetic infections. Clin. Orthop. Relat. Res..

[27] Huang Z., Li W., Lee G.C., Fang X., Xing L., Yang B., Lin J., Zhang W. (2020). Metagenomic next-generation sequencing of synovial fluid demonstrates high accuracy in prosthetic joint infection diagnostics: mNGS for diagnosing PJI. Bone Jt. Res..

[28] Goldberg B., Sichtig H., Geyer C., Ledeboer N., Weinstock G.M. (2015). Making the Leap from Research Laboratory to Clinic: Challenges and Opportunities for Next-Generation Sequencing in Infectious Disease Diagnostics. mBio.

[29] Goswami K., Parvizi J. (2020). Culture-negative periprosthetic joint infection: Is there a diagnostic role for next-generation sequencing?. Expert. Rev. Mol. Diagn..

[30] Sambri A., Campanacci D.A., Pala E., Smolle M.A., Donati D.M., van de Sande M.A.J., Vyrva O., Leithner A., Jeys L., Ruggieri P. (2025). Two-stage revision for infection of oncological megaprostheses: A multicentre EMSOS study. Bone Jt. J..

[31] Bian T., Shao H., Zhou Y., Huang Y., Song Y. (2018). Tests for predicting reimplantation success of two-stage revision for periprosthetic joint infection: A systematic review and meta-analysis. Orthop. Traumatol. Surg. Res. OTSR.

[32] Theil C., Moellenbeck B., Schwarze J., Puetzler J., Klingebiel S., Bockholt S., Gosheger G. (2024). Can the Current Thresholds for Synovial Cell Count and Neutrophil Percentage to Diagnose Prosthetic Joint Infection be Applied to Metal-on-Metal Rotating Hinge Total Knee Arthroplasty?. J. Arthroplast..

[33] Hasan K., Racano A., Deheshi B., Farrokhyar F., Wunder J., Ferguson P., Holt G., Schwartz H., Petrisor B., Bhandari M. (2012). Prophylactic antibiotic regimens in tumor surgery (PARITY) survey. BMC Musculoskelet. Disord..

[34] Schneider P., Heels-Ansdell D., Thabane L., Ghert M. (2021). Prophylactic Antibiotic Regimens In Tumor Surgery (PARITY): A multi-center randomized controlled study comparing alternative antibiotic regimens in patients undergoing tumor resections with endoprosthetic replacements-a statistical analysis plan. Trials.

[35] George J., Newman J.M., Caravella J.W., Klika A.K., Barsoum W.K., Higuera C.A. (2017). Predicting Functional Outcomes After Above Knee Amputation for Infected Total Knee Arthroplasty. J. Arthroplast..

[36] Khan Z., Khan Z.A., Zamora T., Gulia A., Lozano-Calderon S.A., Kurisunkal V.J., Jeys L.M., Laitinen M.K., Abad Repiso S., Abdelbary H. (2025). What is debridement, antibiotics, and implant retention in orthopaedic oncology? a global cross-sectional survey of surgeons’ practices and opinions. Bone Jt. Open.

[37] McConoughey S.J., Howlin R., Granger J.F., Manring M.M., Calhoun J.H., Shirtliff M., Kathju S., Stoodley P. (2014). Biofilms in periprosthetic orthopedic infections. Future Microbiol..

[38] McChesney G.R., Al Farii H., Singleterry S., Lewis V.O., Moon B.S., Satcher R.L., Bird J.E., Lin P.P. (2025). Can Periprosthetic Joint Infection of Tumor Prostheses Be Controlled With Debridement, Antibiotics, and Implant Retention?. Clin. Orthop. Relat. Res..

[39] Gonzalez M.R., Acosta J.I., Clunk M.J., Bedi A.D.S., Karczewski D., Newman E.T., Raskin K.A., Lozano-Calderon S.A. (2024). Debridement, Antibiotics, and Implant Retention (DAIR) Plus Offers Similar Periprosthetic Joint Infection Treatment Success Rates to Two-Stage Revision in Oncologic Megaprosthesis. J. Arthroplast..

[40] Lee S.H., Oh J.H., Lee K.S., Yoo K.H., Kim H.S. (2002). Infection after prosthetic reconstruction in limb salvage surgery. Int. Orthop..

[41] Allison D., Huang E., Ahlmann E., Carney S., Wang L., Menendez L. (2014). Peri-Prosthetic Infection in the Orthopedic Tumor Patient. Reconstr. Rev..

[42] Sigmund I.K., Gamper J., Weber C., Holinka J., Panotopoulos J., Funovics P.T., Windhager R. (2018). Efficacy of different revision procedures for infected megaprostheses in musculoskeletal tumour surgery of the lower limb. PLoS ONE.

[43] Sukhonthamarn K., Tan T.L., Strony J., Brown S., Nazarian D., Parvizi J. (2021). The Fate of Periprosthetic Joint Infection Following Megaprosthesis Reconstruction. JB JS Open Access.

[44] Holzer G., Windhager R., Kotz R. (1997). One-stage revision surgery for infected megaprostheses. J. Bone Jt. Surg. Br. Vol..

[45] Funovics P.T., Hipfl C., Hofstaetter J.G., Puchner S., Kotz R.I., Dominkus M. (2011). Management of septic complications following modular endoprosthetic reconstruction of the proximal femur. Int. Orthop..

[46] Flint M.N., Griffin A.M., Bell R.S., Wunder J.S., Ferguson P.C. (2007). Two-stage revision of infected uncemented lower extremity tumor endoprostheses. J. Arthroplast..

[47] Grimer R.J., Belthur M., Chandrasekar C., Carter S.R., Tillman R.M. (2002). Two-stage revision for infected endoprostheses used in tumor surgery. Clin. Orthop. Relat. Res..

[48] Gonzalez M.R., Pretell-Mazzini J., Lozano-Calderon S.A. (2023). Risk Factors and Management of Prosthetic Joint Infections in Megaprostheses-A Review of the Literature. Antibiotics.

[49] Papadopoulos A., Ribera A., Mavrogenis A.F., Rodriguez-Pardo D., Bonnet E., Salles M.J., Dolores Del Toro M., Nguyen S., Blanco-García A., Skaliczki G. (2019). Multidrug-resistant and extensively drug-resistant Gram-negative prosthetic joint infections: Role of surgery and impact of colistin administration. Int. J. Antimicrob. Agents.

[50] Argenson J.N., Arndt M., Babis G., Battenberg A., Budhiparama N., Catani F., Chen F., de Beaubien B., Ebied A., Esposito S. (2019). Hip and Knee Section, Treatment, Debridement and Retention of Implant: Proceedings of International Consensus on Orthopedic Infections. J. Arthroplast..

[51] van Meurs S.J., Gawlitta D., Heemstra K.A., Poolman R.W., Vogely H.C., Kruyt M.C. (2014). Selection of an optimal antiseptic solution for intraoperative irrigation: An in vitro study. J. Bone Jt. Surg. Am. Vol..

[52] Jeys L., Botello E., Boyle R.A., Ebeid W., Houdek M.T., Kurisunkal V.J., Morgan-Jones R., Morris G.V., Puri A., Ruggieri P. (2025). A modified Delphi consensus on periprosthetic infection in orthopaedic oncology: A report from the Birmingham Orthopaedic Oncology Meeting (BOOM). Bone Jt. J..

[53] Trampuz A., Zimmerli W. (2008). Diagnosis and treatment of implant-associated septic arthritis and osteomyelitis. Curr. Infect. Dis. Rep..

[54] Warth L.C., Hadley C.J., Grossman E.L. (2020). Two-Stage Treatment for Total Knee Arthroplasty Infection Utilizing an Articulating Prefabricated Antibiotic Spacer. J. Arthroplast..

[55] Cooper H.J., Della Valle C.J. (2013). The two-stage standard in revision total hip replacement. Bone Jt. J..

[56] Vasarhelyi E., Sidhu S.P., Somerville L., Lanting B., Naudie D., Howard J. (2022). Static vs Articulating Spacers for Two-Stage Revision Total Knee Arthroplasty: Minimum Five-Year Review. Arthroplast. Today.

[57] Winkler T., Stuhlert M.G.W., Lieb E., Müller M., von Roth P., Preininger B., Trampuz A., Perka C.F. (2019). Outcome of short versus long interval in two-stage exchange for periprosthetic joint infection: A prospective cohort study. Arch. Orthop. Trauma. Surg..

[58] Aali Rezaie A., Goswami K., Shohat N., Tokarski A.T., White A.E., Parvizi J. (2018). Time to Reimplantation: Waiting Longer Confers No Added Benefit. J. Arthroplast..

[59] Sabry F.Y., Buller L., Ahmed S., Klika A.K., Barsoum W.K. (2014). Preoperative prediction of failure following two-stage revision for knee prosthetic joint infections. J. Arthroplast..

[60] Babis G.C., Sakellariou V.I., Pantos P.G., Sasalos G.G., Stavropoulos N.A. (2015). Two-Stage Revision Protocol in Multidrug Resistant Periprosthetic Infection Following Total Hip Arthroplasty Using a Long Interval Between Stages. J. Arthroplast..

[61] Puetzler J., Hofschneider M., Gosheger G., Theil C., Schulze M., Schwarze J., Koch R., Moellenbeck B. (2024). Evaluation of time to reimplantation as a risk factor in two-stage revision with static spacers for periprosthetic knee joint infection. J. Orthop. Traumatol..

[62] Salgado C.D., Dash S., Cantey J.R., Marculescu C.E. (2007). Higher risk of failure of methicillin-resistant *Staphylococcus aureus* prosthetic joint infections. Clin. Orthop. Relat. Res..

[63] Ippolito J.A., Thomson J.E., Rivero S.M., Beebe K.S., Patterson F.R., Benevenia J. (2021). Management of Large Segmental Bone Defects at the Knee With Intramedullary Stabilized Antibiotic Spacers During Two-Stage Treatment of Endoprosthetic Joint Infection. J. Arthroplast..

[64] Nucci N., Gazendam A., Gouveia K., Ghert M., Wilson D. (2020). Management of infected extremity endoprostheses: A systematic review. Eur. J. Orthop. Surg. Traumatol. Orthop. Traumatol..

[65] Hardes J., von Eiff C., Streitbuerger A., Balke M., Budny T., Henrichs M.P., Hauschild G., Ahrens H. (2010). Reduction of periprosthetic infection with silver-coated megaprostheses in patients with bone sarcoma. J. Surg. Oncol..

[66] Politano A.D., Campbell K.T., Rosenberger L.H., Sawyer R.G. (2013). Use of silver in the prevention and treatment of infections: Silver review. Surg. Infect..

[67] Schmidt-Braekling T., Streitbuerger A., Gosheger G., Boettner F., Nottrott M., Ahrens H., Dieckmann R., Guder W., Andreou D., Hauschild G. (2017). Silver-coated megaprostheses: Review of the literature. Eur. J. Orthop. Surg. Traumatol. Orthop. Traumatol..

